# Genomic Database Analysis of Uterine Leiomyosarcoma Mutational Profile

**DOI:** 10.3390/cancers12082126

**Published:** 2020-07-31

**Authors:** Annalisa Astolfi, Margherita Nannini, Valentina Indio, Angela Schipani, Alessandro Rizzo, Anna Myriam Perrone, Pierandrea De Iaco, Maria Giulia Pirini, Antonio De Leo, Milena Urbini, Paola Secchiero, Maria Abbondanza Pantaleo

**Affiliations:** 1 Department of Morphology, Surgery and Experimental Medicine, University of Ferrara, 44121 Ferrara, Italy; annalisa.astolfi@unife.it (A.A.); paola.secchiero@unife.it (P.S.); 2 Medical Oncology Unit, S.Orsola-Malpighi University Hospital, 40138 Bologna, Italy; 3 “Giorgio Prodi” Cancer Research Center, University of Bologna, 40138 Bologna, Italy; valentina.indio2@unibo.it (V.I.); angela.schipani2@unibo.it (A.S.); maria.pantaleo@unibo.it (M.A.P.); 4 Department of Experimental, Diagnostic and Specialty Medicine, S.Orsola-Malpighi University Hospital, University of Bologna, 40138 Bologna, Italy; rizzo.alessandro179@gmail.com; 5 Gynecologic Oncology Unit, S.Orsola-Malpighi University Hospital, 40138 Bologna, Italy; myriam.perrone@aosp.bo.it (A.M.P.); pierandrea.deiaco@unibo.it (P.D.I.); 6 Pathology Unit, S.Orsola-Malpighi University Hospital, 40138 Bologna, Italy; mariagiulia.pirini@aosp.bo.it (M.G.P.); antonio.deleo@unibo.it (A.D.L.); 7 Biosciences Laboratory, Istituto Scientifico Romagnolo per lo Studio e la Cura dei Tumori (IRST) IRCCS, 47014 Meldola, Italy; milena.urbini@irst.emr.it

**Keywords:** uterine leiomyosarcoma, uLMS, genome analysis, *TP53*, *ATRX*, *RB1*, *PTEN*

## Abstract

Uterine Leiomyosarcoma (uLMS) is by far the most common type of uterine sarcoma, characterized by an aggressive clinical course, a heterogeneous genetic profile and a very scarce response to cytotoxic chemotherapy. The genetic make-up of uLMS is an area of active study that could provide essential cues for the development of new therapeutic approaches. A total of 216 patients with uLMS from cBioPortal and AACR-GENIE databases were included in the study. The vast majority of patients (81%) carried at least one mutation in either *TP53*, *RB1*, *ATRX* or *PTEN*. The most frequently mutated gene was *TP53*, with 61% of the patients harboring at least one mutation, followed by *RB1* at 48%. *PTEN* alteration was more frequent in metastases than in primary lesions, consistent with a later acquisition during tumor progression. There was a significant trend for *TP53* and *RB1* mutations to occur together, while both *TP53* and *RB1* were mutually exclusive with respect to *CDKN2A/B* inactivation. Overall survival did not show significant correlation with the mutational status, even if *RB1* mutation emerged as a favorable prognostic factor in the *TP53*-mutant subgroup. This comprehensive analysis shows that uLMS is driven almost exclusively by the inactivation of tumor suppressor genes and suggests that future therapeutic strategies should be directed at targeting the main genetic drivers of uLMS oncogenesis.

## 1. Introduction

Uterine Leiomyosarcomas (uLMS) are rare aggressive malignancies that account for approximately 1% of female genital tumors, with a median age at diagnosis of about 55 years [1,2,3]. LMS is by far the most common type of uterine sarcoma, with an annual incidence of less than two women per 100,000, based on the population-based Surveillance, Epidemiology and End Results (SEER) database [4].

Uterine Leiomyosarcoma are characterized by an aggressive clinical course, a heterogeneous genetic profile and a very poor response to cytotoxic chemotherapy [1,2,3]. The rarity, heterogeneity and peculiar drug-resistance lead to a difficult clinical management of these tumors. 

Surgery remains the gold standard treatment for early stage uLMS, but the risk of recurrence is estimated around 50 to 70% [3,5,6]. Even though several drugs demonstrated clinical activity in advanced or metastatic setting, the role of postoperative adjuvant therapy in uLMS in order to reduce the risk of recurrence remains controversial [7,8]. In the advanced or metastatic uLMS, chemotherapy is considered the standard approach [9,10,11]. Finally, immunotherapy that represents the most recent and impressive hope in cancer treatment failed to show activity in sarcoma and specifically in the leiomyosarcoma histotype [12]. Therefore, uLMS prognosis remains poor regardless of initial stage at diagnosis, while surgery and adjuvant therapy has not been effective at improving the clinical course.

Many cytotoxic regimens have been tested, and the majority of studies have used doxorubicin, ifosfamide, gemcitabine, docetaxel, trabectedin, dacarbazine and pazopanib as single agents or in combination. The response rate by chemotherapy ranges approximatively from 10 to 50%, better especially for combination regimes, but the prognosis of this disease still remains poor [13]. Therefore, the discovery of novel, more effective targeted treatments on the basis of molecular profiling together with the identification of prognostic molecular markers remains an unmet clinical need. 

Currently, studies on genomic characterization of uLMS have been reported, pointing to a tumor type with a low somatic mutational burden when compared to epithelial cancers but carrying distinctive genomic signatures deriving from recurrent mutations in only few genes, widespread DNA copy number alterations and a rather specific gene expression and methylation profile [14,15,16,17]. Genomic profiling is also instrumental for clinical assessment, such as for ctDNA detection for prediction of progressive disease course [18]. A recent study on uterine sarcomas was performed, showing that potentially actionable mutations were identified in nearly half of the analyzed patients, even if the study confirmed the recurrent mutational profile of uLMS dictated by *TP53*, *RB1* and *ATRX* genetic hits [19]. Even if novel genetic signatures are discovered and proposed to stratify patients with respect to prognosis [20], their impact on the clinical management of patients or on the development of novel therapeutic opportunities is still marginal. 

The present study aims to comprehensively catalogue and analyze all available genomic data in public datasets to increase the current knowledge and statistical power on the molecular profile of uLMS in order to identify meaningful molecular markers predictive of disease course and novel potential pharmacological targets. 

## 2. Methods 

### 2.1. Clinical and Mutational Database

Data regarding mutational profiles, copy number alterations and patients’ survival time and status in patients affected by uterine leiomyosarcomas were downloaded from the cBioPortal for Cancer Genomics Database, an open access genomic data portal that is publicly available at http://www.cbioportal.org, and from the AACR-GENIE project [21,22,23]. uLMS data were available in the most recent release of Memorial Sloan Kettering Cancer Center MSK-Impact dataset (MSK) [19], in The Cancer Genome Atlas TCGA Firehose legacy series (TCGA) [15] and in the metastatic solid cancer project from the University of Michigan (UMich) [24], for a total number of patients of 119. Moreover, other samples were available from the AACR-GENIE project [23], and specifically 51 samples from the MSK dataset (MSK/Genie), 39 samples from the Dana-Farber Cancer Institute (DFCI) and 7 from the Vanderbilt-Ingram Cancer Center (VICC). Overall, the number of uLMS samples taken into account was 216. Available clinical data encompassed diagnosis age, fraction of genome altered, metastatic disease status, metastasis site, overall survival time and status. The fraction of genome altered was defined as the length of segments with log2 copy number value larger than 0.2 divided by the length of all segments measured. 

Clinical and genomic data were merged according to the unique patient ID, while mutational and copy number data were restricted to the gene list of the 341 cancer genes sampled in the MSK-Impact study for data consistency (Table S1). The restriction to this set of genes allows us to focus specifically on cancer-related genes and to make the frequency of data coming from whole exome experiments comparable throughout the whole dataset. Mutation data were referred to the OncoKB knowledge base (https://oncokb.org/) for disease-specific levels of evidence of the actionability of mutant alleles, DNA copy number alterations and translocations. For copy number data, only deep deletions (homozygous loss) and amplifications were considered. No statements of approval or informed consent were required for this study since data were obtained from an open access database. A table summarizing clinicopathological data and the associated mutational profiles is shown in Table S2.

### 2.2. Statistical Methods

Categorical data were analyzed using the Fisher’s exact test, and the odds ratio was assessed for the association. Benjamini and Hochberg correction for multiple tests was applied for q-value calculation. Continuous variables were compared by means of two-tailed Student’s t test. To ensure that mutational and copy number data were comparable, we performed a logistic PCA (R package logisticPCA) specifically developed for dimensionality reduction of binary data. We estimated and compared the survival curves using Kaplan–Meier estimation of overall survival and log-rank tests. Cox proportional hazards model was used to analyze associations between molecular data and patient survival. Overall survival (OS) data were obtained from the cBioPortal database directly [21]. Statistical analyses were performed using SPSS (IBM SPSS statistics 25.0, Armonk, NY, USA). All statistical tests were two-sided, and the *p*-value < 0.05 was considered statistically significant. Graph-pad Prism 8.0 (Graph Pad, San Diego, CA, USA) or Oncoprinter tool (https://www.cbioportal.org/oncoprinter) were used to visualize results. 

## 3. Results 

**Figure 1:**
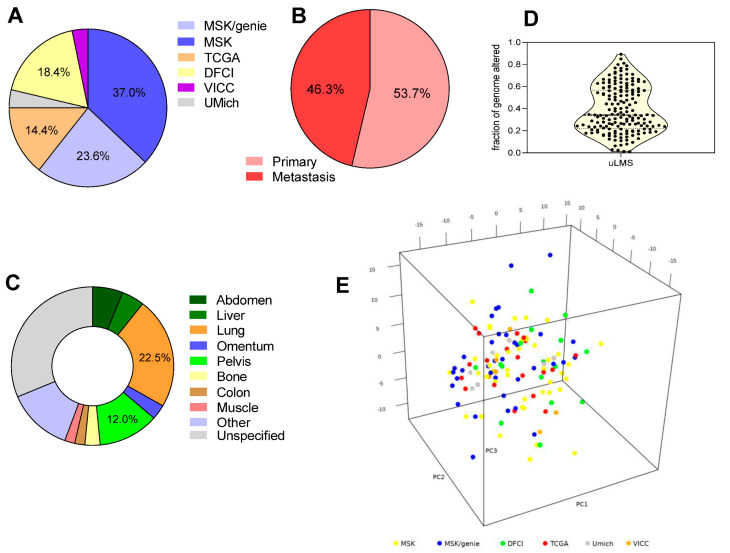
Description of the cohort included in the study. (**A**) Distribution of the samples in the six datasets: MSK, Memorial Sloan Kettering MSK-Impact dataset; MSK/Genie, MSK samples from AACR-GENIE project; TCGA, The Cancer Genome Atlas Firehose legacy; DFCI, Dana-Farber Cancer Institute; VICC, the Vanderbilt-Ingram Cancer Center; UMich, University of Michigan metastatic solid cancer project. (**B**) Origin of the analyzed samples between primary and metastatic sites. (**C**) Distribution of the sites of metastases in the patients with metastatic disease. (**D**) Fraction of genome altered values in the cohort, defined as the length of segments with log2 copy number value larger than 0.2 divided by the length of all segments measured. (**E**) Logistic PCA of binary mutational and copy number data showing that no cluster linked to the different data source is present.

A total of 216 cases of uterine uLMS available from cBioportal database and AACR-GENIE project [21,22,23] were included in the study, of which 80 were from the MSK-Impact dataset [19], 31 from the TCGA Firehose legacy [15], 51 from the MSK/Genie dataset, 39 from the Dana-Farber Cancer Institute (DFCI), 8 from the University of Michigan (UMich) metastatic solid cancer project [24] and 7 from the Vanderbilt-Ingram Cancer Center (VICC) (Figure 1A). 

While TCGA and UMich sequencing data came from whole exome sequencing, those from the MSK center (representing collectively 60% of the whole sample series) came from all-exon targeted sequencing of 341–410 genes; therefore, the combined analysis was referred only to the shared 341 gene signature for consistency. Clinical and sequencing data included analyses half from tissue of primary tumors (54%) and half of metastases (46%) (Figure 1B). Patients enrolled in the studies were mostly with advanced-stage disease in that metastatic disease was confirmed in 133 patients (62%). Sites of metastases were predominantly the lung (22.5%), followed by the pelvis (12.0%), abdomen (6.0%) and liver (4.5%) (Figure 1C). Median age at diagnosis was 56 years (interquartile range (IQR) = 51–63 years). To assess the genomic instability, the fraction of genome altered was taken into account, showing a homogeneous profile across samples, with an average of 0.39 ± 0.02 (Figure 1D). Collectively, median overall survival was 63.96 ± 11.2 months, as shown in previously published data [19], and consistent with a series of patients with mostly advanced disease. No statistically significant difference in overall survival was evident between the two major studies reporting survival data (MSK and TCGA), thus supporting the comparability of the merged dataset. 

**Figure 2:**
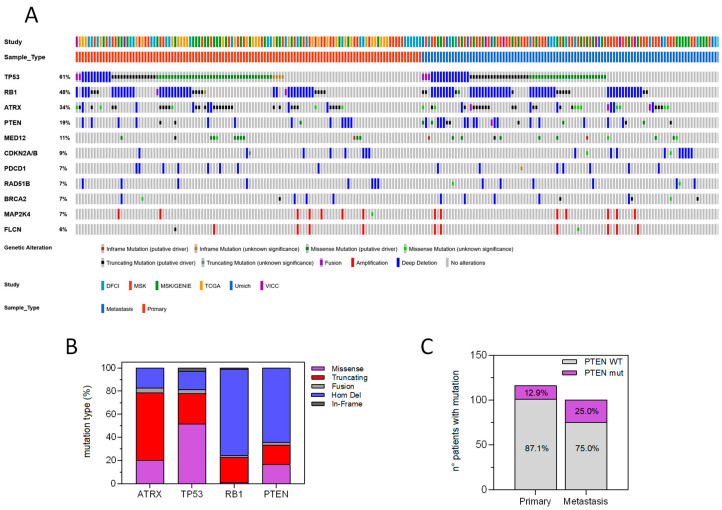
Mutational profile of uLMS. (**A**) Oncoprint representation of the genomic alterations in the cohort stratified by data origin and biopsy site. All alterations are shown, with indication of the OncoKB annotation. (**B**) Distribution of mutation type of the four most frequent genetic lesions in uLMS. (**C**) Association between *PTEN* mutations and tumor sample origin from primary tumor or metastatic site (*p* = 0.023).

Considering sequencing results, we combined mutational profiles with copy number data, taking into account all nonsynonymous variants (nonsense and frameshift, missense, in-frame Ins/del), gene fusions or intragenic rearrangements and homozygous deletions or gene amplifications. To ensure that the data were effectively comparable, we performed a logistic PCA developed for dimensionality reduction of binary data. By means of 3D projections (Figure 1E) we can assess that no cluster linked to the different source of data was present. The analysis showed that the vast majority of the patients carried at least one disrupting mutation in either one of the four recurrently mutated genes, *TP53*, *RB1*, *ATRX* and *PTEN*, with only 19% of the total patient series not harboring at least one mutant allele in these tumor suppressor genes (Figure 2A). More specifically, *TP53* was the most frequently mutated, with 61% of the patients having at least one pathogenic somatic mutation, immediately followed by *RB1* with 48% of patients carrying inactivating mutations or homozygous deletions. *ATRX* and *PTEN* were also well represented, with 34% and 19% of uLMS patients showing at least one mutation or deletion in either one of the two (Figure 2A). 

Mutational spectrum was different, since *TP53* showed mainly missense point mutations (51% of variants) scattered throughout the coding sequence, with just peak recurrence on hotspot codons 248, 173 and 337, followed by truncating mutations (26% of patients with a mutant allele, including nonsense, frameshift or splice-site mutations). Conversely *RB1* and *PTEN* were mainly inactivated by homozygous deletions (75% and 64% of patients with gene alteration, respectively), while *ATRX* was predominantly knocked-out by truncating mutations (59%) (Figure 2B). Other genes were much less hit by mutations, since *CDKN2A/B* homozygous deletions and *MED12* missense mutations were both retrieved in roughly 10% of uLMS patients (Figure 2A). *MED12* mutations were associated with an older age at diagnosis (*p* = 0.034), and a higher fraction of genome altered (*p* = 0.031), suggesting that *MED12*-mutant uLMS represents a different disease, both at the clinical and genomic level. Interestingly, there was a significant tendency for *TP53* and *RB1* mutations to occur together (*q*-value = 0.012), while both *TP53* and *RB1* were mutually exclusive with respect to *CDKN2A/B* inactivation (*q*-value = 0.012 and 0.022, respectively). *TP53*, *ATRX* and *RB1* mutations were equally represented in primary tumors and metastases, while *PTEN* alterations were more frequently observed in metastases than in primary lesions (*p* = 0.023), consistent with a later acquisition during tumor progression (Figure 2C).

Even less were the patients harboring *BRCA2* or *RAD51B* inactivating alterations (7%), thus reinforcing the leading role of the three recurrent onco-suppressors (*TP53*, *RB1* and *ATRX*) already pointed out as key drivers of soft tissue sarcoma development, also in the pathogenesis of uLMS [15]. Moreover, it also stressed the fact that uLMS malignant transformation is driven and supported almost exclusively by the inactivation of tumor suppressor genes and not by the gain of function of actionable oncogenes, which poses major challenges for the development of targeted therapeutic approaches. Nonetheless, the percentage of patients carrying mutations in genes implicated in Homologous Recombination Repair (HRR) of DNA double-strand breaks, e.g., *BRCA2*, *RAD51B* and *PTEN*, overall represented 29% of the series, suggesting that a sizeable fraction of patients could display defective HRR, the so-called BRCAness phenotype. 

**Figure 3:**
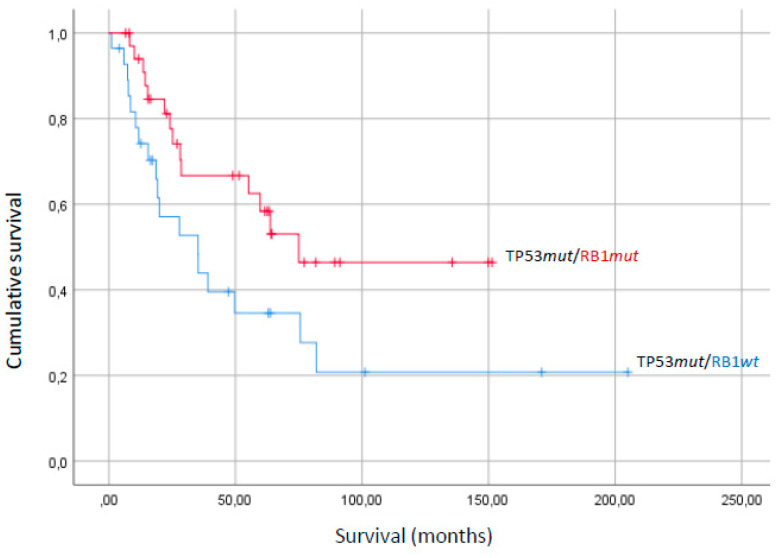
Kaplan–Meier analysis of overall survival of *RB1*-mutant patients vs. *RB1* wild-type among the cohort of *TP53*-mutant patients. (Log-rank *p*-value = 0.04).

Survival analysis was performed on half of the whole patients’ series for whom overall survival time and status was available. Univariate and multivariable analysis of overall survival did not show any significant correlation with the mutational status of either of the four most recurrently altered genes (*TP53*, *RB1*, *ATRX* and *PTEN),* as shown in Table 1. However, due to the sufficiently large series, it was possible to perform survival analysis in subgroups of patients stratified by the two most recurrent alterations, i.e., *TP53* and *RB1*. This way it was possible to recognize *RB1* mutation as a favorable prognostic factor, but only in the *TP53*-mutant patients’ subgroup, since OS of double *TP53mut*/*RB1mut* patients was significantly higher than that of *TP53mut*/*RB1wt* (89.9 vs. 67.1 months) (Figure 3). Conversely, there was a trend, even if with borderline *p*-value, for a better OS of *TP53wt/RB1wt* with respect to *TP53mut/RB1wt* (Table 1). Without suitable cell models of these single and combined mutational events it is difficult to give mechanistic explanations for these results, even if it is tempting to speculate that multiple simultaneous genetic alterations could account for a higher sensitivity of uLMS to chemotherapy or for a more efficient immunological recognition. 

## 4. Discussion

This study reports the integration of molecular data of uLMS deposited in the public molecular cBioPortal for Cancer Genomics dataset and in the AACR-GENIE data portal through a comprehensive analysis of the genomic profile of this disease, of the most frequent genetic lesions and their association with clinical features, reporting genetic data from 216 uLMS samples. Up to now this is the largest study on the uLMS genetic profile reported so far, whose relevance mainly resides on the statistical power to confirm the recurrence of mutational signatures and uncover associations with patients’ clinical data. Our study confirmed that the key mutant genes characterizing uLMS are *TP53*, *RB1*, *ATRX* and *PTEN*, which collectively account for 81% of the analyzed tumor samples, a fact that was already anticipated in previous studies [14,15,16,17] and in the recent Memorial Sloan Kettering study on uterine sarcomas [19], whose data were used in this analysis altogether with other data. Of relevance, due to a higher number of cases analyzed, our study confirmed that *PTEN* loss is an event predominantly occurring in metastases instead of in primary tumors [19], thus suggesting that *PTEN* mutations are supposedly acquired later during tumor progression. This result reinforces the view in which the main players in uLMS pathogenesis are *TP53*, *RB1* and *ATRX*, which are genes involved in the cell cycle, genome stability and chromatin remodeling. In particular *ATRX* loss is intimately linked to the alternative lengthening of telomeres (ALT), a mechanism that contributes to cellular immortality in cancer, genetic instability and increased mutational rate [25,26]. The loss of function of *TP53*, “the genome guardian” that controls cell cycle and DNA integrity [27] or *ATRX*, likely accounts for the hallmarks of uLMS, such as the widespread genomic imbalances [28]. This scenario depicting a primary role for *TP53* and *ATRX* in uLMS tumorigenesis is further supported by a recent study performed in a zebrafish model showing that heterozygous loss of *ATRX* in the context of *TP53*/*NF1* deficiency induces the onset of multiple tumors, particularly of sarcomas [29]. Furthermore, the high proportion of patients carrying *TP53* genetic hits likely stimulates other considerations on the window of therapeutic opportunities in uLMS, since *TP53* mutations have been associated with resistance to several cytotoxic therapies including drugs used for patients with sarcomas, such as anthracyclines or antimetabolites [30]. The recurrent onco-suppressors genes involved in uLMS including *BRCA2*, coupled with the frequent inactivation of genes implicated in DNA damage and homologous recombination repair, further depict this disease as a tumor with defects in proper DNA repair and reinforce the role of PARP inhibitors in the treatment of uLMS [31]. Indeed, Seligson and coworkers have already shown that HRR pathway alterations are enriched in uLMS by means of *BRCA2* loss and that PARP inhibitors demonstrated durable clinical benefit in uLMS patients with *BRCA2* inactivation [32]. Furthermore, Hensley et al. reported clinical benefit in all five *BRCA2*-mutant uLMS patients treated with PARP inhibitors, further supporting the potential actionability of HRR alterations in uterine leiomyosarcoma [19]. Actually, a phase II clinical trial investigating PARP-inhibitors in uterine LMS is currently ongoing (ClinicalTrials.gov Identifier: NCT03880019). Lastly, it would be interesting to analyze the putative contribution of germline mutations in uLMS, since in other sarcomas harboring the same genetic alterations a large-scale analysis showed a significant frequency of germline pathogenic variants [33]. 

Concerning other less frequent mutations, it is important to observe that MED12 mutations are rare (11%), with striking difference with respect to benign leiomyomas, which are reported to carry *MED12* exon 2 mutations in roughly 70% of cases [34]. Interestingly, previous reports showed slightly higher frequency of *MED12* mutations in uLMS, a difference that could be linked to the lower number of cases analyzed [14,17] but maybe also to the random selection of the subset of tumors arising from previous leiomyomas, whose clinical features still have to be uncovered. Our analysis supported this view in which uLMS arise independently from previous benign tumors and represent a different disease, both at the clinical and genomic level. 

Even though these molecular findings have been largely evaluated and confirmed in our comprehensive genomic database analysis, no molecular marker was demonstrated to have a clinical impact and can be fully considered in practice. The USA and European guidelines do not incorporate molecular markers in the workup for diagnosis and treatments in uLMS [9,35]. In some clinical settings of uLMS in which the management is still controversial, for example the patients who may benefit from adjuvant chemotherapy after surgery of primary tumors, the identification of molecular markers could help the physicians in the decision-making process for treatment choice. Therefore, since whole genomic studies in uLMS have been largely completed, in the future genomic studies should be more focused on small selected populations of patients to address specific medical needs. Up until now, in 2020, the standard medical treatment in uLMS does not include any targeted approach, being still founded on chemotherapy, and the standard first-line treatment is still represented by anthracyclines [9,10,11]. In sarcoma setting, trabectedin only may act through the inhibition of aberrant transcription factors of the translocated histotypes, but in the case of uLMS it acts as a standard alkylating agent [36]. Finally, pazopanib acts as a multi-tyrosine kinase inhibitor and through the anti-angiogenic effect rather than by the inhibition of a specific molecular driver [37]. Finally, probably due to low somatic mutational burden, immunotherapy does not represent a new challenge and hope in the leiomyosarcoma histotype [12], even if a thorough description of the tumor microenvironment could provide novel data on molecules involved in angiogenesis or immunosuppression that might be relevant new targets in uLMS and could identify the subgroup of patients that would benefit from this approach.

These results emphasize further that personalized treatments for uLMS cannot overlook anymore the role of the major genetic drivers of uLMS reported here and in previous studies to deliver an effective and targeted approach. Preclinical studies should now provide reliable cell models of uLMS to mimic tumor progression and identify synthetic-lethal combinations that target recurrently mutated pathways and that could be exploited as therapeutic targets. 

## 5. Conclusions

In summary, this database analysis of the mutational profile of 216 cases of uLMS confirms that the most frequent alterations involve *TP53*, *RB1* and *ATRX*, and shows that *PTEN* mutations tend to be acquired at advanced disease stages, that *TP53* mutations co-occur with *RB1*, that both are mutually exclusive with *CDKN2A/B*, and that *MED12* mutations characterize patients with an older age at diagnosis and a higher fraction of altered genome. Moreover, a significant proportion of patients carry putative *BRCA*-related mutations, possibly representing a selected population showing benefit from PARP inhibitors treatment. This large-scale database analysis can support the design of appropriate uLMS preclinical models to devise novel pharmacological approaches targeted to interfere with the main uLMS genetic drivers.

## Figures and Tables

**Table 1:** Univariate Kaplan–Meier survival analyses in uLMS cohort.

Subgroup	Class	Average OS (Months)	SE	Log-Rank *p*-Value
All	*TP53* wild-type	117.9	20.5	0.18
	*TP53* mutant	92.1	12.7	
	*RB1* wild-type	89.2	12.9	0.22
	*RB1* mutant	131.4	17.7	
	*ATRX* wild-type	111.4	15.1	0.63
	*ATRX* mutant	101.9	15.9	
	*PTEN* wild-type	93.3	15.1	0.52
	*PTEN* mutant	146.9	15.9	
*TP53* mutant	*RB1* wild-type	67.1	16.4	0.04
	*RB1* mutant	89.9	11.8	
*RB1* wild-type	*TP53* wild-type	99.2	14.4	0.05
	*TP53* mutant	67.1	16.4	

## Supplementary Materials

The following are available online at https://www.mdpi.com/2072-6694/12/8/2126/s1, Table S1: List of genes analyzed in the study, Table S2: Clinicopathological and mutational data of the 216 uLMS patients.
